# Experimentally induced cartilage degeneration treated by pulsed electromagnetic field stimulation; an in vitro study on bovine cartilage

**DOI:** 10.1186/s12891-015-0760-6

**Published:** 2015-10-20

**Authors:** Francesca Veronesi, Milena Fini, Gianluca Giavaresi, Alessia Ongaro, Monica De Mattei, Agnese Pellati, Stefania Setti, Matilde Tschon

**Affiliations:** Department Rizzoli RIT, Rizzoli Orthopedic Institute, Laboratory of Biocompatibility, Innovative Technologies and Advanced Therapies, Bologna, 40136 Italy; Laboratory of Preclinical and Surgical Studies, Rizzoli Orthopedic Institute, Bologna, 40136 Italy; Department of Morphology, Surgery and Experimental Medicine, University of Ferrara, Ferrara, 44121 Italy; IGEA – Clinical Biophysic, Carpi (Modena), 41012 Italy

**Keywords:** Osteoarthritis, Cartilage, Explants, Inflammation, Microenvironment, Pulsed electromagnetic field

## Abstract

**Background:**

Osteoarthritis (OA) is the final result of progressive alterations to articular cartilage structure, composition and cellularity, followed by an increase in the concentration of pro-inflammatory cytokines in joint synovial fluid. Even though the effect of pulsed electromagnetic field (PEMF) stimulation in counteracting OA progression and inflammation is of increasing interest, because of its anabolic and anti-inflammatory properties, the present study aimed to improve the knowledge on cartilage extracellular matrix (ECM) and chondrocyte changes related to the exposure of PEMF, from a histological and histomorphometric point of view.

**Methods:**

An *in vitro* OA model was realized, culturing bovine cartilage explants with a high dose of interleukin 1β (IL1β, 50 ng/ml) at different experimental times (24 h, and 7 and 21 days). The effects of PEMFs (75 Hz, 1.5 mT) were evaluated in cartilage explants treated with IL1β or not (control), in terms of cartilage structure, cellularity and proteoglycans, glycosaminoglycans, collagen II and transforming growth factor β1 synthesis by using histology, histomorphometry and immunohistochemistry.

**Results:**

Making a comparison with control cartilage, IL1β-treated explants showed a decrease in cartilage matrix, structure and cellularity parameters. PEMFs were able to counteract the progression of OA acting on both cartilage cellularity and ECM in cartilage previously treated with IL1β. Normal distribution (Kolmogroc-Smirnov test) and homoscedasticity (Levene test) of data were verified, then, the non-parametric Kruskal Wallis test followed by Mann-Whiteny *U* test for pairwise comparisons were performed. The *p*-value was adjusted according to the Dunn-Sidak correction.

**Conclusions:**

These results, obtained by culturing and treating cartilage explants from two different joints, confirmed that PEMF stimulation can be used as adjuvant therapy to preserve cartilage from detrimental effects of high inflammatory cytokine levels during OA.

## Background

The presence of pro-inflammatory cytokines in the joint microenvironment favors cartilage degeneration and osteoarthritis (OA) progression with alterations of proteoglycans (PGs) and collagen fibers, prevalently collagen type II (Coll II) [1]. Among pro-inflammatory cytokines, interleukin1β (IL1β) plays a pivotal role in inducing degradation of extracellular matrix (ECM) components and synthesis of other pro-inflammatory cytokines, chemokines and proteolitic enzymes. Since cartilage has a poor intrinsic reparative capability if not treated, OA leads to progressive disability and requirement of joint arthroplasty [2].

Current therapeutic strategies to prevent OA include the use of nonsteroidal anti-inflammatory drugs (NSAIDs) [3], that act at articular and not cellular levels, intra-articular injection of hyaluronic acid (HA) and physical exercises [4], that act only in the muscle of the joint. These treatments relieve pain and inflammation and improve functionality, but do not resolve the pathological process once triggered, thus the outcomes are inconclusive and remain as long as these therapies are administered. For this reason, orthopedic research investigates chondroprotective treatments able to reduce the local inflammatory microenvironment and to favor damaged articular cartilage anabolic activity.

Pulsed electromagnetic field (PEMF) stimulation has been already studied and proposed for the regeneration of musculoskeletal tissues such as cartilage [5], bone [6, 7], tendon [8] and ligament [9]. As far as cartilage tissue is concerned, PEMFs positively affect chondrocyte proliferation and ECM component synthesis [10]. However, the existing studies, performed with different PEMF physical parameters and exposure times, gave contradictory results [11–15], in terms of chondrocyte viability, DNA content, glycosaminoglycans (GAGs), Coll II, PGs and aggrecan gene expression and production. In a previous *in vitro* study, on bovine cartilage explants, we conducted a dose–response study to find the optimal dosage, in terms of PEMF frequency and exposure intensity, able to stimulate significantly PG synthesis [16]. Furthermore, the stimulatory effect of PEMFs on PG synthesis in presence of IL1β and in combination with insulin-like growth factor 1 (IGF-I) was observed [17, 18]. PEMF and IGF-I showed an additive effect on PG synthesis in human OA cartilage explants cultured in the absence or presence of IL1β [19]. In *in vivo* studies, PEMF stimulation was able to limit the progression of OA of increasing severity [20–22], to reduce IL1β and tumor necrosis factor-α (TNF-α) concentrations and to increase transforming growth factor-β1 (TGFβ1) in the synovial fluid of sheep treated with autologous osteochondral grafts [23]. In clinical studies, PEMF stimulation was successfully used to treat pain, knee swelling and functionality, after total knee arthroplasty [24, 25] and in early knee OA patients [26]. In other studies, beneficial symptomatic effects [27, 28] or additional effects to physical treatments [29] were not observed in OA patients.

Even if PEMFs act on the joint microenvironment and have anabolic and chondroprotective actions, the mechanisms by which PEMFs exert their effects on biological systems remain not completely understood. To our knowledge, no histological and histomorphometric evaluations have assessed the effects of PEMF stimulation in an inflammatory OA microenvironment obtained *in vitro* with the addition of a high IL1β dose.

The present study aimed to analyze whether PEMF stimulation (75 Hz, 1.5 mT), applied to bovine cartilage explants, derived from two different joints of the same animals, was able to counteract the catabolic effect of a high dose of IL1β. In comparison to 2D monolayer chondrocyte cultures, the cartilaginous 3D explants cultured with IL1β are a well-accepted method to measure and evaluate the effect and mechanism of action of a therapy, by simultaneously evaluating chondrocytes and cartilage matrix and by closely mimicking the clinical situation of OA joint, in which a high dose of IL1β is found in the early stages of OA [30] and its concentration can be further increased by surgical intervention [2].

It was hypothesized that the PEMF stimulation alone, without the addition of other biological stimuli, might improve the production of the most important cartilage ECM components (PGs, GAGs, COLL II) and anabolic factor (TGF-β1) and preserve cartilage structure, thus controlling OA development. To demonstrate this hypothesis it was adopted an already set and validated *in vitro* model of OA [18, 31], which used a high dose of IL1β (50 ng/ml) to create an inflammatory OA microenvironment. After 24 h, 7 and 21 days of culture, PEMF effect, in combination or not with IL1β, was evaluated through histological, histomorphometric and immunohistochemical analyses. In this study it was observed that PEMFs were able to counteract the progression of OA acting on both cartilage cellularity and ECM in cartilage previously treated with IL1β.

## Methods

### Cartilage explant cultures and treatment conditions

Full-thickness explants of bovine articular cartilage were aseptically dissected, by using a 4 mm dermal punch (Stiefel Laboratories, Milan, Italy), from the metacarpophalangeal(MC) and metatarsophalangeal (MT) joints of 14-18-month-old animals (Limousine breed), as previously described [16–18]. The abattoir gave permission for the use of cartilage explants in this study and no ethical approval, from a recognized ethics committee, was required for this study. The cartilage was explanted shortly after the bovine slaughter and cartilage discs (three discs in each well) were cultured in 0.5 ml culture medium in multiwells (Nunc, Denmark, 1.6 cm the diameter of each well). Before PEMF exposure and IL1β administration, all explants were allowed to equilibrate in culture for 48 h in DMEM/F12 (Life Technologies, Monza, Italy) supplemented with 10 % FBS and antibiotics (penicillin 100 units/ml, streptomycin 0.1 mg/ml) (complete medium) and for an additional 48 h in medium without serum, at 37 °C in an atmosphere of 5 % CO_2_. During the experiments, explants were cultured in complete medium in the absence and presence of IL1β (50 ng/ml) [18]. Half cultures were exposed to PEMF throughout the entire culture period (24 h, 7 and 21 days). Medium was changed at the beginning of the exposure (time 0) and every 3 days.

For each experimental time, 4 experimental groups were set up and each group was composed of 3 explant discs from MC and 3 more from MT joints:

- Control group (CTR): cartilage explants;
- IL1β group: cartilage explants submitted to IL1β administration;
- PEMF group: cartilage explants exposed to PEMFs;
- IL1β + PEMF group: cartilage explants submitted to IL1β and exposed to PEMFs.

### Characteristics of PEMFs and exposure conditions

The PEMF generator system was the same as that used in previous studies [9, 16–18, 32, 33]. It consisted of a pair of circular Helmoltz coils of copper wire placed opposite to each other and in a signal generator (Igea S.p.A., Carpi, Italy). The multiwell plates were placed between this pair of Helmoltz coils, so that the plane of the coils was perpendicular to the multiwell plates, and the direction of the induced electric field was perpendicular to the direction of the magnetic field. The power generator produced a pulsed signal with the following parameters: the pulse duration was 1.3 ms and the frequency was 75Hz, yielding a duty cycle of 1/10. The intensity peak of the magnetic field was 1.5mT and the induced electric field, as detected with a standard coil probe (50 turns, 0.5 cm internal diameter of the coil probe, 0.2 copper diameter), was 0.07 mV/cm.

The intensity of the magnetic field was detected between two coils from one side to the other, by the Hall probe of the Gaussmeter (LE, Gaussmeter DG500, USA), with a reading sensitivity of 0.2 %. Inside this area, where the multiwell plate was placed, the magnetic field was uniform. Cartilage explants were exposed to continuous PEMF for 24 h, 7 or 21 days. PEMF unexposed cultures were placed inside the same incubator at a distance where no difference from background magnetic field was observed when the PEMF generator was turned on. In each experiment, controls and treatments were performed in triplicate wells.

### Histology

At the end of the experimental times, cartilage explants, cultured in the above mentioned conditions, were routinely processed for paraffin embedding procedures. Briefly, samples were fixed for 24 h in 10 % neutral buffered formalin solution in PBS, extensively rinsed in distilled water, dehydrated in graded alcohol solutions (70, 95 %, two times and 100 %, three times, one hour for each step), cleared in xylene e finally paraffin embedded. Sections (5 ± 1 μm) were cut along the longitudinal axis of the samples by a semi-automated microtome (Microm H340E, Germany) and stained with Toluidine Blue, for the PG quantification and Safranin O-Fast green staining, for the GAG quantification. Three non-consecutive sections for each sample were evaluated, by 2 independent histologists through a semi-quantitative score (modified O’Driscoll score) [34]. The modified O’Driscoll score (Table 1: minimum 0 = osteoarthritic cartilage - maximum 11 = normal intact cartilage) analyzed 4 cartilage aspects: Safranin O staining, surface regularity, cellularity and chondrocyte clustering.

**Table 1:** Modified O’Driscoll score

Parameter	Feature	Grade
Safranin O staining	Normal or nearly normal	3
	Moderate	2
	Slight	1
	None	0
Surface regularity	Smooth and intact	3
	Superficial horizontal lamination	2
	Fissures 25–100 % of the thickness	1
	Severe disruption, including fibrillation	0
Cellularity	Normal cellularity	3
	Slight hypocellularity	2
	Moderate hypocellularity	1
	Severe hypocellularity	0
Chondrocyte clustering	No clusters	2
	<25 % of the cells	1
	25–100 % of the cells	0

### Immunohistochemical stainings (TGFβ1 and Coll II)

Cartilage sections were dewaxed in decreasing graded ethanol solutions until PBS rinsing for 20 min and then immunostained for TGFβ1 and Coll II. Briefly, after fixation, sections were extensively rinsed in PBS and permeabilized by incubation in 0.3 % hydrogen peroxide in PBS solution for 15 min. For Coll II immunostaining, sections were pre-treated for antigen unmasking with 0.2 % Pronase (P-8811, Sigma, Mo, USA) solution in PBS for 30 min at 37 °C. Then, 10 % normal serum was added for 1 h at room temperature to block nonspecific antibody binding and the primary antibodies (rabbit polyclonal antibody anti TGFβ1, sc-146 and mouse monoclonal antibody anti collagen II, sc-52658, 1:50 dilutions, Santa Cruz Biotechnology, CA, USA) were applied and incubated overnight at 4 °C. After rinsing in PBS, slides were incubated with appropriate biotinylated secondary antibody and with horseradish peroxidase-streptavidin complex for 1 h each (ABC Staining System, Biotechnology, CA, USA). Sample reaction was developed with 3,3-diaminobenzidine substrate and permanently mounted. Negative controls, by omitting the primary antibody, were included to check proper specificity and performance of the applied reagents.

### Histomorphometric measurements

Slices stained with Toluidine Blue or Safranin O-Fast Green and immunostained for TGFβ1 or Coll II were observed by a light microscope (BX51, Olympus Italia Srl, Segrate-Milano, Italy) and three Regions of Interest (ROI) for each slide were grabbed at a 40× magnification. Image analysis by Leica Q-Win Software (Leica Microsystem, Wetzlar, Germany) was performed and the threshold applied was One-dimensional (LUTs: Lookup Table Transforms) based on selected ranges of RGB channels to measure the following parameters expressed as percentage:

- GAGs, as the ratio between the Safranin O stained areas and the total ROI areas;
- PGs, as the ratio between the Toluidine Blue stained areas and the total ROI areas;
- TGFβ1, as the ratio between the immunopositive stained areas and the total ROI areas;
- Coll II, as the ratio between the immunopositive stained areas and the total ROI areas.

### Statistical analysis

Statistical analysis was performed using the IBM SPSS Statistics v.21.0 software (IBM Corp, USA). Firstly, normal distribution (Kolmogroc-Smirnov test) and homoscedasticity (Levene test) of data were verified, then, the non-parametric Kruskal Wallis test followed by Mann-Whiteny *U* test for pairwise comparisons were performed. The *P*-value was adjusted according to the Dunn-Sidak correction [adj *p* = 1 - (1 – *p*)^k^, where *k* is the number of pairwise comparisons]. Since the preliminary Kruskal-Wallis test by distinguish MC and MP joints did not show significant differences between the two harvesting sites within each type of treatment, data were analyzed when merged.

## Results

### The *in vitro* cartilage OA model after IL1β administration

**Fig. 1:**
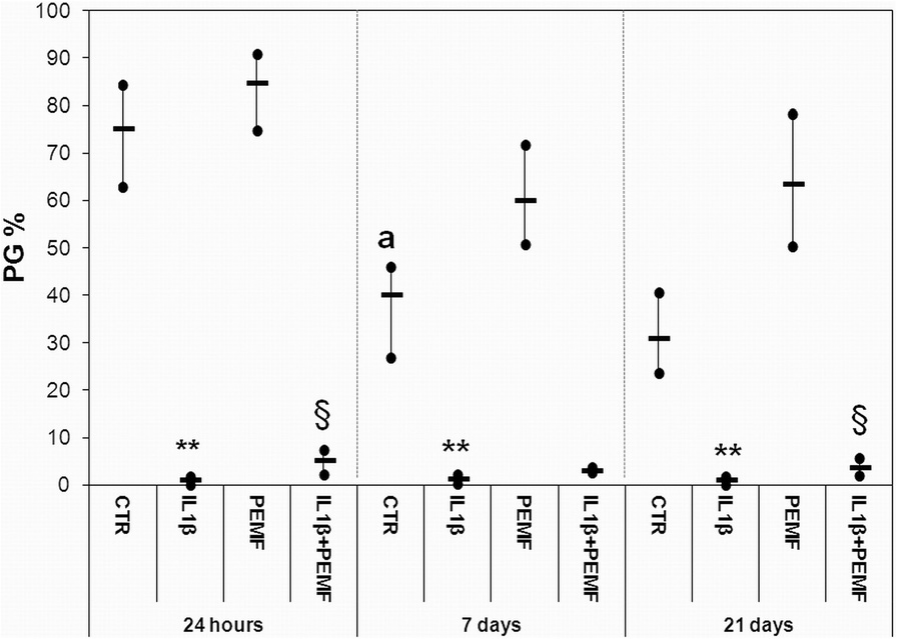
Boxplots of histomorphometric measurements of PGs expressed as percentage in bovine cartilage explants. Black line = median; extreme values = minimum-maximum. Control (CTR), exposed to PEMF (PEMF), treated with IL1β (IL1β) or treated with IL1β and stimulated with PEMF (IL1β + PEMF) explants. 24 h, 7 and 21 days (*n* = 6). Mann-Whitney *U* test: - Within each experimental time: IL1β *versus* CTR (**, *p* < 0.005); IL1β + PEMF *versus* IL1β (§, *p* < 0.05); - Within each group: 7 days *versus* 24 h (^a^, *p* < 0.05)

**Fig. 2:**
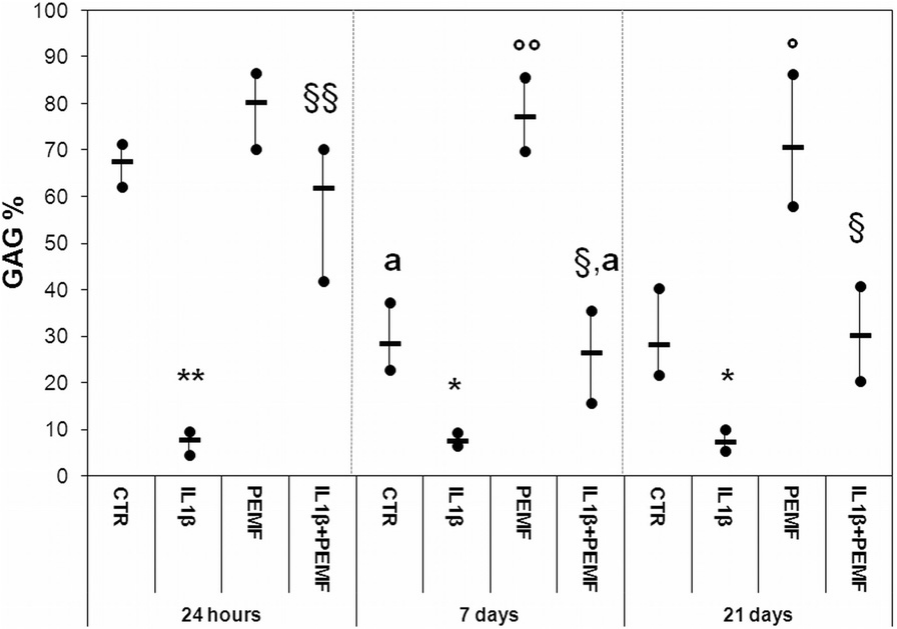
Boxplots of histomorphometric measurements of GAGs expressed as percentage in bovine cartilage explants. Black line = median; extreme values = minimum-maximum. Control (CTR), exposed to PEMF (PEMF), treated with IL1β (IL1β) or treated with IL1β and stimulated with PEMF (IL1β + PEMF) explants. 24 h, 7 and 21 days (*n* = 6). Mann-Whitney *U* test: - Within each experimental time: IL1β *versus* CTR (*, *p* < 0.05; **, *p* < 0.005); PEMF *versus *CTR (°, *p* < 0.05; °°, *p* < 0.005); IL1β + PEMF* versus* IL1β (§, *p* < 0.05; §§, *p* < 0.005); - Within each group: 7 days *versus* 24 h (^a^, *p* < 0.05)

**Fig. 3:**
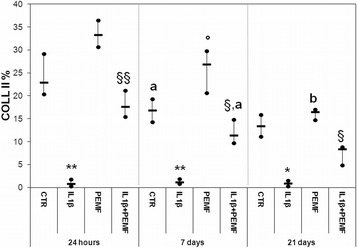
Boxplots of histomorphometric measurements of Coll II expressed as percentage in bovine cartilage explants. Black line = median; extreme values = minimum-maximum. Control (CTR), exposed to PEMF (PEMF), treated with IL1β (IL1β) or treated with IL1β and stimulated with PEMF (IL1β + PEMF) explants. 24 h, 7 and 21 days (*n* = 6). Mann-Whitney *U* test: - Within each experimental time: IL1β *versus* CTR (*, *p* < 0.05; **, *p* < 0.005); PEMF *versus* CTR (°, *p* < 0.05); IL1β + PEMF *versus* IL1β (§, *p* < 0.05; §§, *p* < 0.005); - Within each group: 7 days *versus* 24 h (^a^, *p* < 0.05); 21 days *versus* 7 days (^b^, *p* < 0.005)

In the CTR Group the production of PGs, GAGs and COLL II significantly decreased at 7 days in comparison with cultures at 24 h by −47 % (*p* = 0.022), −58 % (*p* = 0.008) and −27 % (*p* = 0.038), respectively (Figs. 1, 2, 3).

**Fig. 4:**
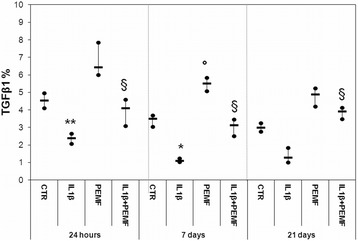
Boxplots of histomorphometric measurements of TGF-β1 expressed as percentage in bovine cartilage explants. Black line = median; extreme values = minimum-maximum. Control (CTR), exposed to PEMF (PEMF), treated with IL1β (IL1β) or treated with IL1β and stimulated with PEMF (IL1β + PEMF) explants. 24 h, 7 and 21 days (*n* = 6). Mann-Whitney *U* test: - Within each experimental time: IL1β *versus* CTR (*, *p* < 0.05; **, *p* < 0.005); PEMF *versus* CTR (°, *p* < 0.05); IL1β + PEMF *versus* IL1β (§, *p* < 0.05)

**Fig. 5:**
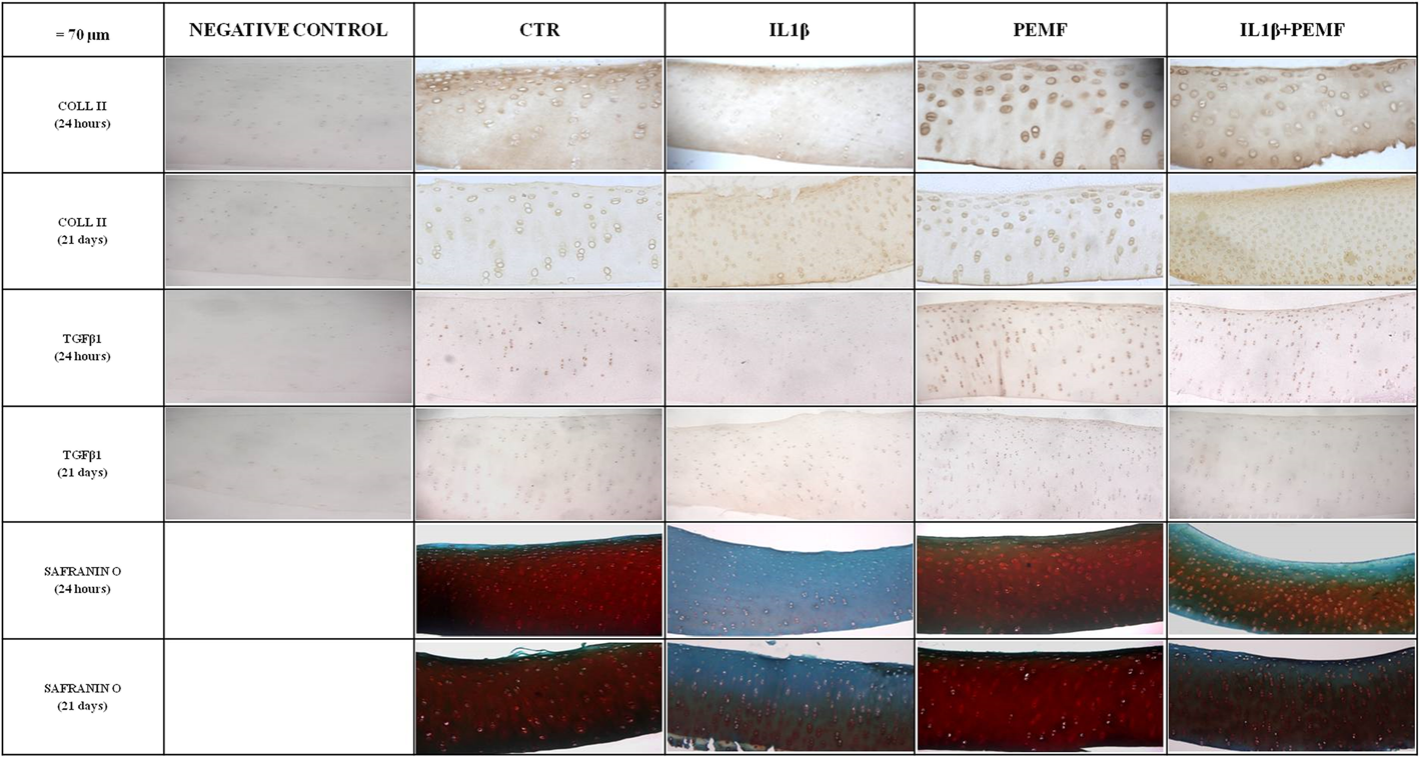
Immunohistochemical and histological images of bovine cartilage explants. Control (CTR), exposed to PEMF (PEMF), treated with IL1β (IL1β) or treated with IL1β and stimulated with PEMF (IL1β + PEMF) explants. 24 h and 21 days. Magnification at 20x. Bar = 70 μm. Negative control column contains 4 images of the negative control of the relative markers (COLL II and TGFβ1)

As expected, the treatment with IL1β significantly decreased the PGs, GAGs, Coll II and TGFβ1 production in cartilage explants as compared to CTR at each experimental time, except for TGFβ1 at 21 days (Figs. 1, 2, 3, 4). Specifically, PGs decreased by 96–99 % (at 24 h *p* = 0.0005, at 7 days *p* = 0.0005 and at 21 days *p* = 0.0005), GAGs by 88 % at 24 h (*p* = 0.0005) and about 73 % at 7 (*p* = 0.008) and 21 (*p* = 0.03) days and Coll II by 92–96 % (at 24 h *p* = 0.0005, at 7 days *p* = 0.0005 and at 21 days *p* = 0.04) (Figs. 1, 2, 3). The production of TGFβ1 significantly decreased at 24 h (*p* = 0.0005, 47 %) and 7 days (*p* = 0.017, 68 %) (Fig. 4). Figure 5 shows the immunohistochemical and histological images of explants stained for Coll II, TGFβ1 and Safranin O at 24 h and 21 days.

**Fig. 6:**
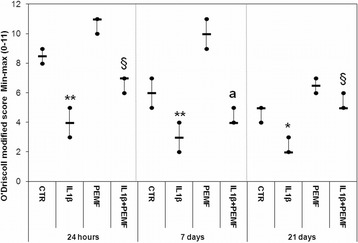
Boxplots of total O’Driscoll modified score in bovine cartilage explants. Black line = median; extreme values = minimum-maximum. Control (CTR), exposed to PEMF (PEMF), treated with IL1β (IL1β) or treated with IL1β and stimulated with PEMF (IL1β + PEMF) explants. 24 h, 7 and 21 days (*n* = 6). Mann-Whitney *U* test: - Within each experimental time: IL1β *versus* CTR (*, *p* < 0.05; **, *p* < 0.005); IL1β + PEMF *versus* IL1β (§, *p* < 0.05); - Within each group: 7 days *versus* 24 h (^a^, *p* < 0.05)

Because of the IL1β effect, the O’Driscoll score progressively worsened by 50–60 %, during the experimental times, in comparison to CTR (at 24 h *p* = 0.0005, at 7 days *p* = 0.0005 and at 21 days *p* = 0.04) (Fig. 6). By analyzing each score parameter (Table 2), it was noted that, the cellularity (*p* = 0.025) and chondrocyte clustering (*p* = 0.025) decreased significantly by 50 % in the CTR group from 7 to 21 days and from 24 h to 7 days, respectively. At 24 h, the reduction induced by IL1β on each O’Driscoll score parameter in comparison with CTR was 50 % for Safranin-O staining (*p* = 0.0005), surface regularity (*p* = 0.002) and cellularity (*p* = 0.0005) and 75 % for chondrocyte clustering (*p* = 0.0005), whereas at 7 and 21 days it was 50 % for surface regularity (at 7 days *p* = 0.0005 and at 21 days *p* = 0.0005), 50–100 % for cellularity (at 7 days *p* = 0.001 and at 21 days *p* = 0.001) and 100 % for chondrocyte clustering (at 7 days *p* = 0.001 and at 21 days *p* = 0.02).

**Table 2:** Median values (minimum and maximum values) of O’Driscoll modified score parameters in cartilage explants

Experimental time	O’driscoll score parameters	CTR	IL1β	PEMF	IL1β + PEMF
24 h	Safranin-O staining	2 (2–3)	1 (1–2)**	3 (2–3)	2 (2–2)^§^
	Surface regularity	2 (2–3)	1 (1–2)**	3 (3–3)	2 (2–2)
	Chondrocyte clustering	2 (1–2)	0.5 (0–1)**	2 (2–2)	1 (1–2)
	Cellularity	2 (2–3)	1 (1–1)**	3 (2–3)	2 (1–2)^§§^
7 days	Safranin-O staining	1 (1–2)	0 (0–1)	3 (2–3)°°	1 (1–2)^§^
	Surface regularity	2 (1–3)	1 (0–1)**	3 (2–3)	1 (1–2)
	Chondrocyte clustering	1 (1–1)	0 (0–1)**^,a^	2 (1–2)°°	1 (1–2)^§^
	Cellularity	2 (1–2)	1 (0–1)**	2 (2–3)	1 (1–2)
21 days	Safranin-O staining	1 (1–2)	0 (0–1)	2 (2–2)°	1 (1–1)^§§^
	Surface regularity	2 (1–2)	1 (1–1)**	2 (2–2)	2 (1–2)^§§, b^
	Chondrocyte clustering	1 (1–1)	0 (0–0)*	1 (1–1)^b^	1 (1–2)^§^
	Cellularity	1 (1–1)	0 (0–1)**^,a^	1.5 (1–2)	1 (1–1)^§§^

Control (CTR), exposed to PEMF (PEMF), treated with IL1β (IL1β) or treated with IL1β and stimulated with PEMF (IL1β + PEMF) explants. 24 h, 7 and 21 days (*n* = 6)

Mann-Whitney *U* test: - Within each experimental time: IL1β *versus* CTR (*, *p* < 0.05; **, *p* < 0.005); PEMF *versus* CTR (°, *p* < 0.05; °°, *p* < 0.005); IL1β + PEMF *versus* IL1β (§, *p* < 0.05; §§, *p* < 0.005); Within each group: 7 days *versus* 24 h (^a^, *p* < 0.05); 21 days *versus* 7 days (^a^, *p* < 0.05; ^b^, *p* < 0.005)

### Effects of PEMF stimulation

As also observed by histology in Fig. 5, in bovine cartilage explants stimulated by PEMF, no significant increases in PGs were found in comparison with CTR explants at each experimental time (Fig. 1). Conversely, PEMF stimulated GAG production at 7 (*p* = 0.0005, 170 %) and 21 (*p* = 0.005, 151 %) days, as well as Coll II (*p* = 0.005, 59 %) and TGFβ1 (*p* = 0.005, 58 %) at 7 days (Figs. 2, 3, 4). By analyzing data over time, Coll II in the PEMF group diminished from 7 to 21 days (*p* = 0.004, 38 %).

When bovine cartilage explants were cultured with IL1β and were stimulated with PEMFs, very high increases in PG production compared to the IL1β group were observed at 24 h (*p* = 0.01, 439 %) and 21 days (*p* = 0.04, 235 %) (Fig. 1). The IL1β + PEMF group also showed increases in GAG production in comparison to the IL1β group at 24 h (*p* = 0.0005, 684 %), 7 (*p* = 0.02, 250 %) and 21 days (*p* = 0.01, 307 %) (Fig. 2). Coll II increased massively in the IL1β + PEMF group at 24 h (*p* = 0.002, 1890 %), 7 (*p* = 0.02, 835 %) and 21 days (*p* = 0.007, 734 %) in comparison to IL1β (Fig. 3). GAG and Coll II synthesis decreased by 57 % (*p* = 0.02) and 35 % (*p* = 0.02), respectively, from 24 h to 7 days in the IL1β + PEMF group. Regarding TGFβ1 production, it increased progressively at all experimental times (at 24 h *p* = 0.005, 71 %, at 7 days *p* = 0.02, 184 % and at 21 days *p* = 0.007, 204 %) in comparison to the IL1β group (Fig. 4).

The O’Driscoll score results of the PEMF-treated group did not differ from those of the CTR group at each experimental time (Fig. 6). Taking into account each O’Driscoll score parameter (Table 2), PEMF stimulation improved chondrocyte clustering at 7 days (*p* = 0.0005, 100 %) and Safranin-O staining at 7 (*p* = 0.0005, 100 %) and 21 days (*p* = 0.03, 200 %) compared to that of the CTR group.

The effect of PEMF stimulation, applied to IL1β-treated cartilage explants (IL1β + PEMF group), improved the O’Driscoll score in comparison to the IL1β group, at both 24 h (*p* = 0.02, 75 %) and 21 days (*p* = 0.03, 150 %) (Fig. 6). A decrease in O’Driscoll score was highlighted between 24 h and 7 days in the IL1β + PEMF group (*p* = 0.007, 43 %). The IL1β + PEMF group improved by about 100 % for Safranin-O staining (*p* = 0.02) and cellularity (*p* = 0.003) at 24 h, for chondrocyte clustering (*p* = 0.008) and Safranin-O staining (*p* = 0.008) at 7 days, and for surface regularity (*p* = 0.003), Safranin-O staining (*p* = 0.001), chondrocyte clustering (*p* = 0.005) and cellularity (*p* = 0.001) at 21 days compared to those of the IL1β group (Table 2). In addition, surface regularity of the IL1β + PEMF group decreased by 50 % (*p* = 0.003) from 24 h to 7 days (Table 2).

## Discussion

The present study aimed to evaluate the effects and mechanisms of action of PEMF stimulation (75 Hz, 1.5 mT) in preserving cartilage from OA structural deterioration. The *in vitro* effects were investigated on bovine cartilage explants harvested from two different joints. To simulate the OA microenvironment, a high IL1β concentration (50 ng/ml) was administered in explant cultures, as set in an our previous *in vitro* study [30], for a longer period of 21 days and the changes in the structure and cellularity of CTR and IL1β-treated cartilage explants, either exposed or not to PEMFs, were analyzed. The concentration of IL-1β found in the synovial fluid of patients affected by OA is between 0.068 and 0.33 pg/ml [35]. However, most of the *in vitro* literature studies, that aimed to re-create an inflammatory microenvironment, added 10 ng/ml of IL-1β in the medium of cartilage explants [36–38], while there are other authors that employed 50 or 100 ng/ml [39–41]. The so high concentration of IL-1β used in the present study was chosen to employed an intermediate cytokine dosage and to evaluate if PEMF stimulation is able to restore cartilage ECM biological and morphological properties also with a huge concentration of IL-1β than the most employed one (10 ng/ml). The current data confirmed the hypotheses of the study. The addition of a high dose of IL1β to the culture medium induced a significant reduction, not only in structural parameters, but also ECM components and TGFβ1 synthesis, a decline observed at all experimental times until 21 days. These results are in agreement with those of previous *in vitro* studies, that observed GAGs, PGs and Coll II loss with the addition of IL1β in the culture medium of human, bovine and horse cartilage explants [30, 42, 43]. Although the joint inflammatory microenvironment of OA consists of different inflammatory mediators, such as matrix metalloproteinases (MMPs), the disintegrin and metalloproteinases with thrombospondin motifs (ADAMTs) and other pro-inflammatory cytokines, TNF-α and Interleukin-6 (IL-6), data suggested that IL1β might have the strongest effect. Indeed, with the addition of IL1β in the culture medium, the most important components of cartilage ECM and TGFβ1 synthesis were reduced and other cartilage structural parameters (matrix and cellularity) were compromised. Coll II, PGs and GAGs are the most abundant components of cartilage ECM, which are responsible for the ECM organization and give shear and tensile properties and the ability to resist compressive loads to cartilage.

Secondly, the stimulation with PEMFs, applied to healthy explants, did not modify PG content or the structural parameters at any of the experimental times, and moreover, increases in GAGs and Coll II were observed after 24 h of culture. Instead, PEMFs improved all the analyzed ECM proteins and the structural parameters in IL1β-treated explants, also at 21 days of culture. Similar results were published by Brighton et al., in 2006 and 2008 using capacitively coupled electric fields (CCEF). They observed an increase in Coll II and PG production in human and bovine cartilage explants, treated or not with IL1β, after stimulation [44, 45]. Finally, in this study it was also observed that IL1β and PEMFs have an opposite effect on TGFβ1, the anabolic growth factor that induces matrix production, mesenchymal stem cell chondrogenic differentiation and chondrocyte proliferation [46].

This study allowed a more complete view of the role of PEMFs that has been already investigated in previous our *in vitro* studies [18, 31]. The strengths of the study were: 1) the histological and histomorphometric evaluations on *in vitro* cartilage explants, stimulated with PEMF parameters already used in patients suffering from knee pain due to cartilage degeneration [47]; 2) the long-term culture of cartilaginous explants (21 days) and 3) the comparative analysis in two different joints. A limitation of the study was the absence, in the culture explants model, of the subchondral bone, that is recognized to play a role in OA development. It is noteworthy that the score employed in this study is a modification of the score proposed by O’Driscoll SW, et al., in 1988 [34]: only the parameters that regard the matrix, the structure and the chondrocyte hypocellularity and clustering could be evaluated.

In comparison to 2D monolayer chondrocyte cultures and *in vivo* situation, the 3D cartilage explants, adopted in this study, are a more homogenous model to evaluate cartilage metabolism. Chondrocytes are maintained in their physiological microenvironment, the de-differentiation phenomenon is prevented and the inter-individual variables are reduced [48]. In addition, the use of cartilage explants is a more suitable *in vitro* method than chondrocytes embedded in alginate cultures to study PEMF and IL1β effects, because the chondrocytes are embedded in their native ECM. In fact the changes in ECM composition may play an important role in maintaining osteoarthritis damage and the alterations in hyaluronate degradation can be an alternative mechanism for the regulation of proteoglycan release from cartilage following IL1β stimulation [49]. 3D explants are reliable even at long experimental times because the effects of IL1β treatment and PEMF stimulation were evident also at 21 days of culture. Many *in vitro* culture studies evaluated bovine cartilage because of the advantageous easy availability and similar thickness to human cartilage [50]. Moreover, the metabolic activity and structural composition of bovine cartilage is reported to be quite similar to those of humans [50].

## Conclusions

This study shows that the addition of IL1β to the culture medium has a detrimental effect on cartilage explants by reducing PG, GAG, Coll II and TGFβ1 quantification and O’Driscoll modified score parameters. PEMF stimulation is able to counteract these catabolic effects both on ECM and chondrocyte features. It was also found that PEMFs have a greater effect on cartilage explants compromised by IL1β in comparison to normal tissues. These results confirm the role of PEMFs in chondroprotection and, as shown by the long-term results, they suggest their ability to limit OA progression.

## Abbreviations

OA: Osteoarthritis
PEMF: Pulsed electromagnetic field
ECM: Extracellular matrix
IL1β: Interleukin 1β
PGs: Proteoglycans
Coll II: Collagen type II
NSAIDs: Nonsteroidal anti-inflammatory drugs
HA: Hyaluronic acid
GAGs: Glycosaminoglycans
IGF-I: Insulin-like growth factor 1
TNF-α: Tumor necrosis factor-α
TGFβ1: Transforming growth factor-β1
MC: Metacarpophalangeal
MT: Metatarsophalangeal
CTR: Control group
MMPs: Metalloproteinases
ADAMTs: The disintegrin and metalloproteinases with thrombospondin motifs
IL-6: Interleukin-6
CCEF: Capacitively coupled electric fields
